# Discovery of myosin light chain kinase gene variant in a patient with tetralogy of Fallot suffering aortic dissection: Implications for pathogenesis and the role of family and population screening^☆^

**DOI:** 10.1016/j.ijcchd.2024.100544

**Published:** 2024-09-18

**Authors:** Radoslaw Debiec, Armia Ebeid, Stephen Hamby, Odeta Anciunaite, Anne Illsley, Ali Nizam, Madiha Iqbal, Kassem Safwan, Tariq Saifullah, Frances Bu’Lock, Toru Suzuki, Nilesh J. Samani, Tom Webb, Aidan P. Bolger

**Affiliations:** a Department of Cardiovascular Sciences and NIHR Leicester Biomedical Research Centre, University of Leicester, College of Medicine Biological Sciences and Psychology, Glenfield Hospital, Groby Road LE39QP, Leicester, UK; b East Midlands Congenital Heart Centre, University Hospitals of Leicester NHS Trust Glenfield Hospital, Groby Road, LE39Q, Leicester, UK

**Keywords:** Tetralogy of Fallot, Aortic dissection, Myosin light chain kinase

## Abstract

**Background:**

Thoracic aortic dissection (TAD) is an uncommon complication in patients with Tetralogy of Fallot (TOF). Information concerning risk factors for TAD in patients with TOF is very limited.

**Methods:**

We report a case of Stanford type A TAD in a female patient with previously repaired TOF. Whole exome sequencing (WES); Novogene UK, Agilent V6 capture kit, Illumina HiSeq 100x depth) was performed to identify genetic variants in genes known to be associated with TAD. A systematic literature review was performed in the NCBI PubMed database to identify case reports of TAD in patients with TOF.

**Results:**

The patient was a 31-year-old female who developed Stanford type A aortic dissection having had TOF repair at the age of four years. The thoracic aorta was only minimally dilated (sinus of Valsalva 43 mm) on clinical review 16 months prior to TAD. Of note the patient had completed pregnancy 5 months prior to the dissection. There were no other high-risk features predisposing to TAD. WES identified rare genetic variant in a gene previously associated with TAD: *MYLK* (p.Arg1405His). The literature review identified nine other case reports of TAD in patients with TOF. The reported patients, had no clinical characteristics that distinguished them from the wider population of patients with TOF.

**Conclusions:**

The presence of a rare genetic variant in *MYLK* is a plausible explanation for the clinical presentation. The variant will need further verification to confirm pathogenicity. Pathogenic *MYLK* variants have been previously reported in context of dissection with minimally dilated aortas.

## Highlights

• We describe aortic dissection in a patient with tetralogy of Fallot, without any high-risk predisposing characteristics.
• We identified a likely pathogenic variant in the *MYLK* gene as a plausible explanation for this complication.
• Our finding may provide an explanation for why these rare and serious events occur in a common congenital condition.

## 1 Introduction

Tetralogy of Fallot (TOF) is one of the most common complex congenital heart malformations, accounting for approximately 5 % of all congenital heart disease [1]. The lesion comprises a spectrum of sub-valvular, valvular and/or supra-valvular pulmonary stenosis, ventricular septal defect, overriding aorta and right ventricular hypertrophy. Since the first description of surgical repair in 1955 the operating techniques and clinical management have evolved resulting in a progressive reduction in mortality with survival to adulthood improving from 68.5 % to approximately 94 % over the last 50 years [2].

Improved early survival has seen the occurrence of late complications in adult patients, necessitating long term follow up and further therapeutic interventions in this group. Aortic dilatation is commonly seen late after TOF repair. In a large cross-sectional study, clinically relevant dilatation of the aortic root (≥40 mm), in adult patients after TOF repair, was present in approximately one third of patients [3] with clear evidence of a progressive nature [[3], [4], [5]]. Several factors have been associated with increased aortic dimensions including pulmonary atresia type TOF, right sided aortic arch, older age at the time of repair, male sex and presence of aortic regurgitation [3,4].

Histopathological assessments of aortic specimens from patients with TOF haave shown changes typically associated with aortopathy from other causes, including the presence of cystic medial necrosis and elastin fragmentation [6]. Mechanistic studies of aortic stiffness have also confirmed abnormal mechanical properties of the proximal thoracic aorta of patients with TOF irrespective of repair status [7].

Thoracic aortic dissection (TAD) is an uncommon complication in patients with TOF but its true incidence is unknown [8]. A review of hospital admissions of patients with TOF in the USA indicated that of all admissions in this group of patients only 0.06 % (11/18,353) were related to TAD in a period of 14 years [9]. Despite being rare, TAD was associated with 45 % mortality in this group [9].

Information regarding risk factors for TAD in patients with TOF is derived mainly from clinical case reports. To date, these have not included detailed analysis of genetic mutations known to be associated with TAD and so it has been difficult to understand why this highly lethal complication occurs in this cohort. A greater understanding of the genetic basis for TAD in individuals with TOF may help identify those at risk in this group. It could also help identify first degree relatives carrying the same genetic risk allowing for clinical screening and prophylactic surgical intervention. Here we report a case of Stanford type A TAD in a female patient with previously repaired TOF and explore the potential underlying genetic basis for this complication.

## 2 Methods

### 2.1 Case report

A patient with TOF who had suffered TAD was approached for inclusion in this study. Informed consent was obtained for access to the medical notes, imaging studies, operation reports and clinical investigations from The East Midlands Congenital Heart Centre, UK. The patient was consented for blood sampling and whole exome sequencing and research procedures were performed under the NIHR Leicester Biomedical Research Centre - Sample and Data Collection for Cardiovascular Research ethics protocol (BRICCS ethic ref 09/H0406/114, August/2010; The study protocol conforms to the ethical guidelines of the 1975 Declaration of Helsinki). Separate written consent was obtained from the patient for publication of results including use of imaging studies and data from whole exome sequencing.

### 2.2 Whole exome sequencing and genetic analysis

A sample of blood (EDTA) was obtained from the patient and an automated DNA extraction was carried out on the QIAsymphony SP robot using QIAsymphony DSP DNA Midi Kit (QIAGEN GmbH, Hilden, Germany). The sample was checked for purity and normalized to a standard concentration of 100 ng/μL. Whole‐exome sequencing was performed by Novogene UK. Agilent V6 capture kit was used for library capture and sequencing was performed using the Illumina HiSeq platform (Illumina, San Diego, USA). Sequence reads were aligned to the reference genome (GRCh38) using Burrows‐Wheeler Aligner BWA V0.7.15 [10]. Duplicates were marked with Picard and base quality score recalibration was carried out before variant calling with GATK 4.3 [11]. Variant calls were hard filtered according to quality control metrics: Quality by depth, Fisher strand (to detect strand bias), RMS Mapping quality, and read position rank sum (testing for distance from the end of read). Variant annotation was carried out using the Ensembl variant effect predictor (VEP) version 110 [12]. Annotation included the assignment of amino acid changes, gnomAD (Version 4.1.0) minor allele frequency (MAF) and functional prediction using the Combined Annotation Dependent Depletion (CADD) tool [13]. Variant prioritisation was carried out to highlight rare (gnomAD population maximum MAF <0.01) variants in genes listed within the Genomics England gene panels (https://panelapp.genomicsengland.co.uk/) for “Familial non syndromic congenital heart disease” (CHD) (Version 1.86) and “Thoracic aortic aneurysm or dissection” (TAAD) (Version 1.127). Five genes not present in either gene panel, *FOXC1* [14]*, FOXC2* [14], *KDR* [14]*, NDRG4* [15] and *SMARCC2* [15], but which have had rare candidate variants reported for TOF, were also included in our analysis. In silico prediction of variant deleteriousness were assessed in VarSome [16], which compiles a consensus of computational pathogenicity predictions from dbNSFP (v4.7) [17]. Mendelian Inheritance in Man (MIM) numbers and phenotypes were collected from the online browser (http://omim.org/) [18]. Variants were interpreted according to American College of Medical Genetics and Genomics (ACMG) guidelines [19].

### 2.3 Literature search

A systematic literature search was performed independently by two researchers following the PRISMA guidelines in the NCBI PubMed database using the terms: “*dissection tetralogy of Fallot*”, “*acute aortic syndrome tetralogy of Fallot*” and “*dissection ToF*” [20]. The search included all manuscripts published before December 1, 2023. To be eligible the manuscripts had to be published in English, be report of a case, case series or database containing information about patient with TOF and TAD (any type, including iatrogenic). The study team also performed review of bibliography of filtered manuscripts to identify other potential publications.

## 3 Results

### 3.1 Case report

The patient was a female of mixed ethnicity (African and African-Caribbean descent). She was born in the UK at term by normal vaginal delivery and showed slow but steady weight gain. The patient was incidentally found to have a heart murmur at the age of six weeks and at the age of seven months was admitted to hospital after a chest x-ray revealed cardiomegaly and pulmonary plethora. At the time of admission, ECG showed sinus rhythm with electrocardiographic features of right ventricular hypertrophy (QRS axis +90). Cardiac catheter confirmed the diagnosis of TOF: large ventricular septal defect, infundibular pulmonary stenosis, aorta overriding the right ventricle by 60 %, and left-sided aortic arch. Systolic pulmonary artery pressure was 30–35 mmHg, mean of 24 mmHg; 40/0 mmHg in the right ventricle outflow tract compared to 60/0 in the RV body giving a gradient across the right ventricular infundibulum of 20 mmHg. Aortic pressure was 84/55 mmHg. The decision was made to delay surgical treatment at that time.

At the age of 14 months, the patient moved to Zambia before returning to the UK at the age of four years. During the subsequent follow up visits the patient was noted to have gradually worsening dyspnoea and decreased exercise tolerance (no history of squatting, syncope or cyanosis). The patient underwent total correction of TOF including Dacron patch closure of VSD and infundibular resection combined with right ventricular outflow tract enlargement (pulmonary valve annulus was 15 mm with good valve function so no transannular patch was needed). The patient required implantation of permanent epicardial pacemaker due to post-operative complete heart block. The patient remained under regular clinical follow up.

At the age of 31 years, the patient presented to Glenfield Hospital, Leicester, UK with a two-week history of stuttering chest and back pain. On the evening of admission, the patient experienced a severe episode of chest pain and acutely deteriorated. Urgent bedside echocardiogram demonstrated a dissection flap in the proximal ascending aorta and severe aortic regurgitation. CT aortogram demonstrated a dilated aortic root of 45–48 mm with dissection limited to the aortic root and ascending aorta; ending just proximal to the aortic arch; the ascending aorta measured 35 mm at the level of the main pulmonary artery. There was no history of thoracic aortic aneurysm or acute aortic syndrome in first degree relatives.

The patient underwent emergency composite root and ascending aorta replacement with mechanical valve (23 mm Carbomedic composite aortic valve graft). The recovery was complicated by a peri-operative stroke involving the basal ganglia, which resulted in balance and speech problems.

The most recent clinic review had been 16 months prior to admission. At that time the patient was clinically well and normotensive. Echocardiography had demonstrated mild dilatation of the aortic root (sinus of Valsalva 43 mm; 26 mm/m) and normal size of ascending aorta (measurement not available). There was a trileaflet aortic valve with no stenosis and mild central regurgitation. There was no residual VSD. The right ventricle was mildly dilated with good function, there was no significant pulmonary stenosis (Doppler parameters were not recorded in the report). The left ventricle had normal dimensions and function. Of note, the patient had completed pregnancy 5 months prior to the dissection (full term, normal vaginal delivery, no complications).

### 3.2 Results of the whole exome sequencing

Whole exome sequencing revealed the presence of six rare genetic variants (gnomAD population maximum MAF <0.01) in genes present on the Genomics England TAAD or CHD gene panels [21]. The variants are presented in Table 1.

**Table 1:** Rare genetic variants identified through whole exome sequencing.

**Genetic Variant**	**RSID**	**Gene**	**HGVSc**	**HGVSp**	**Gene MIM number**	**Phenotype MIM number**	**MIM phenotype**	**ClinVar**	**MAF**	**CADD**
chr3_123657200_C/T	rs745547260	*MYLK*	c.4214G > A	p.Arg1405His	600922	613780	Aortic aneurysm, familial thoracic 7	Uncertain significance	0.000045	28.5
chr5_129461675_C/T	rs144822778	*ADAMTS19*	c.665C > T	p.Pro222Leu	607513	620067	Cardiac valvular dysplasia 2	–	0.0067	4.2
chr5_180625967_C/G	rs147703852	*FLT4*	c.1323G > C	p.Gln441His	136352	618780/153100	Congenital heart defects, multiple types, 7/Lymphatic malformation 1	–	0.0066	19.6
chr9_130878562_G/A	rs62638716	*ABL1*	c.1418G > A	p.Arg473Gln	189980	617602	Congenital heart defects and skeletal malformations syndrome	Benign	0.0096	23.1
chr15_66703337_A/G	rs374058045	*SMAD6*	c.79A > G	p.Ser27Gly	602931	614823	Aortic valve disease 2	Likely benign	0.0046	9.0
chr20_62817585_C/T	rs745914662	*COL9A3*	c.97C > T	p.Pro33Ser	120270	620022/600969	Stickler syndrome, type VI/Epiphyseal dysplasia, multiple, 3, with or without myopathy	Uncertain significance	0.000059	22.5

RSID - RS identifier of genetic variant, HGVSc – Human Genome Variation Society coding change, HGVSp - Human Genome Variation Society protein change, MIM - Mendelian Inheritance in Man number, MAF - minor allele frequency, gnomAD population maximum, CADD -Combined Annotation Dependent Depletion phred score.

The *MYLK* (myosin light chain kinase) missense variant, rs745547260 (c.4214G > A; p.Arg1405His) alters a conserved amino acid residue within the fibronectin type 3 domain of the MYLK protein and is predicted to be deleterious. Pathogenic variants in *MYLK* are an established but rare cause of TAD with patients usually having little to no aortic dilation before dissection [22]. ClinVar contains four entries for rs745547260 as a variant of uncertain significance, including three submissions for familial TAD, with affected status recorded as unknown, and one submission, where the condition was not provided. Given the rarity, damage prediction and phenotype match, we consider the variant likely pathogenic for the patient's TAD [23].

Heterozygous mutations in *FLT4* (fms related receptor tyrosine kinase 4), which encodes VEGF3 (vascular endothelial growth factor 3) are an established cause of TOF [24]. Most *FLT4* ToF mutations are nonsense or frameshift changes with a smaller number of implicated missense variants [24]. We consider the identified missense change, rs147703852 (c.1323G > C; p.Gln441His), to be likely benign due to a higher allele frequency relative to previously identified *FLT4* and TOF pathogenic variants and benign computational prediction.

We do not consider the other four variants as candidates due to a combination of lack of phenotype correlation, higher allele frequency and benign in silico prediction.

### 3.3 Literature search

Search conducted of the PubMed database December 1, 2023, using the terms “dissection tetralogy of Fallot” & “acute aortic syndrome tetralogy of Fallot”; “dissection TOF”, returned 104, 25, and 277 publications, respectively. Among these, 361 unique references were identified. 345 manuscripts were excluded as not meeting the eligibility criteria based on their titles. The remaining sixteen references were evaluated based on their abstract. Ultimately ten manuscripts met the inclusion criteria. One additional manuscript was added after review of the bibliography of the nine preselected publications. Of the total of 11 manuscripts, one manuscript was a report of a large database of hospital admissions in the US and one was a study on echocardiographic progression of aortic root dilatation in children after TOF repair.

Egbe et al. obtained International Classification of Diseases, Ninth Revision, Clinical Modification (ICD-9-CM) codes from the National Inpatient sample Database and calculated a ratio of admission with Stanford type A aortic dissection diagnosed in patients with TOF to all admissions of patients with TOF over a period of 14 years. TAD accounted for only 0.06 % of all admissions of patients with the diagnosis of TOF. Male sex (OR, 6.91; 95 % CI, 4.85–8.54; P < 0.001), age >60 years (OR, 2.41; 95 % CI, 1.23–3.25; P = 0.013) and hypertension (OR, 1.74; 95 % CI, 1.06–3.19; P = 0.037) were identified as risk factors of TAD in TOF. The diagnosis was associated with significant mortality 5/11 (45 %). The information on aortic dimensions, aortic valve morphology and function or genetic investigations were not available.

Grotenhuis et al. described a longitudinal analysis of echocardiographic progression of aortic root dilatation in 768 children with TOF and described a single case of aortic dissection [25]. This occurred in a 9-month-old with an aortic annulus of 1.56 cm (Z score 4.8) and no aortic regurgitation. The results of the genetic analysis were reported as normal,

Clinical details of the remaining nine case reports identified through the literature search are presented in Table 2 [[26], [27], [28], [29], [30], [31], [32], [33], [34]]. Two reports [30,33] described patients with more complex anatomical variants that may be considered outlier with the more typical TOF anatomy seen in the patient reported here. The remaining seven reports ([[26], [27], [28], [29],^31^,^32^,^34^]) describe single cases of TAD in exclusively male patients between the ages of 8–61 years. Of note, two of these individuals (Vaikhunt et al. and Shehata et al.) had undergone aortic valve replacement at, 23 years (Vaikhunt et al., indication not stated) and approximately one year (Shehata et al., aortic regurgitation) prior to TAD. There was limited information on the aortic dimensions at last follow up before TAD. The minimum reported sinus of Valsalva (SoV) was 51 mm and maximum 53 mm with the largest diameter of aorta measured at 70 mm (site of measurement not stated). The aortic dimensions measured after admission with TAD ranged from 55 mm to 79.6 mm for SoV and 70.5 mm–93 mm for the ascending aorta, respectively. The patient with uncorrected TOF reported by Haruki et al. showed evidence of rapidly progressing aortic dilatation with change in the diameter of ascending aorta from 48 mm to 55 mm within two years prior to dissection.

**Table 2:** Clinical details of patients with aortic dissection and tetralogy of Fallot identified through literature search.

Author/year/PMID	Sex (M/F)	Ethnicity	Detailed congenital diagnosis	Age the time of repair/type of repair	Associated features	Genetic investigations	Size of aorta during last follow up before dissection	Age at the time of aortic dissection (years)	Size of aorta at the time of dissection	Type and extend of dissection	Type of repair	Histology
Kim et al., /2005/15907429	M	NA	NA	21 years/preserved pulmonary valve	Residual VSD post repair	NA	NA	30	SoV - 64.5 mm, AscAo - 70.5 mm	Stanford Type A dissection from ascending aorta to common iliac arteries	Bentall procedure involving a composite conduit with a mechanical valvular prosthesis	No evidence of cystic medial degeneration
Rathi et al., /2005/15860407	M	Caucasian	Presence of bronchial fistulas communicating with left atrium	6 months/several repairs (no further details)	Severe biventricular dilatation	NA	NA	36	Sov - 61 mm, AscAo - 93 × 83mm	Stanford Type A dissection extending to aortic arch	NA	NA
Konstantinov et al., /2010/20674941	M	NA	NA	2 months/no details	Tall stature, scoliosis, aberrant right subclavian artery, mild aortic regurgitation, mild sub-valvular aortic stenosis, mild residual pulmonary regurgitation	22q11 deletion	60 × 70 mm (exact site of measurement not stated)	18	NA	Stanford type A dissection extending from ascending aorta to right common carotid artery	Valve-sparing aortic root replacement with a 30-mm Valsalva graft (Vascutek) and resection of sub-aortic stenosis.	NA
Wijesekera et al.,/2014/24794962	M	NA	NA	14	NA	NA	Sov - 53 mm, AscAo - 49 mm	60	SoV - 55 mm	Stanford Type A dissection - limited dissection of the right coronary sinus	Bentall procedure	NA
Jariwala et al., /2017/28622727	M	NA	Large (1.78 cm) maligned peri-membranous ventricular septal defect with overriding of the aorta, and severe right ventricular outflow tract obstruction at the level of the infundibulum with a pressure gradient of 75 mm Hg	Unrepaired	Clubbing, cyanosis and elevated haemoglobin 19.2 g/L; mild aortic valve regurgitation, left ventricular apical thrombus	NA	NA	30	NA	Stanford Type A dissection from SoV to descending aorta	VSD closure with dacron patch through right atrium, infundibular resection, 22-mm interposition graft in the ascending aorta with securing of the proximal and distal end with Teflon felt continuing into the true lumen.	NA
Chow et al., /2020/33014199	M	Chinese	NA	4 years, Infundibular resection via high transverse right ventriculotomy and closure of ventricular septal defect	The two cMRI performed at 29 years old and 32 years old respectively revealed mild dilation of right ventricle, satisfactory right ventricular systolic function, mild pulmonary regurgitation and mild interval growth of aortic root dilation from 4.8 cm to 5.1 cm	22q11 - excluded, no familial history of dissection	Sov - 51 mm (3 years before dissection)	35	Sov - 79.6 mm, AscAo - 76 × 88mm	Stanford type A dissection extending from SoV to descending aorta (Th9 level)	Bentall procedure	Myxoid degeneration and focal calcification of aortic valve. There was mild cystic medial degeneration of the aortic wall
Vaikhunt et al., /2022/35615213	M	NA	NA	5	Aortic valve replacement with mechanical prosthesis at the age of 38 years; single coronary artery arising from the left coronary sinus with a retro-aortic course of the right coronary artery; obesity, hypertension, previous haemorrhagic stroke and retinal artery occlusion	NA	SoV - NA, AscAo - 69 mm (10 days prior to another scan showing dissection)	61	NA	Stanford type A dissection extending from SoV to aortic arch	Aortic root replacement with bio-root: 29-mm Edwards Lifesciences and Ispiris Resilia aortic valve sewn inside a 32-mm Dacron graft). The single coronary artery was reimplanted; A total aortic arch replacement with branched prosthesis was performed	NA
Haruki et al., /2023/37521580	F	NA	Pulmonary atresia, severely hypoplastic pulmonary arteries, major aorto-pulmonary collaterals	Unrepaired	No familial history of aortic disease. Patent PDA	22q11 excluded, Identified genetic variant in *FBN1* Pro1128Ala	AscAo - 55 mm, descending aorta 45 mm; nine months before dissection., There was evidence of progressive dilatation of the ascending aorta (48 mm at the age of 44 years and 55 mm at the age of 46 years)	46	Descending aorta 48 mm	Stanford type B aortic dissection	Conservative management	NA
Shehata et al., /2023/38152233	M	NA	NA	3	Hypertension, smoking, depression. Re-operated at the age of 54 years: closure of residual VSD, aortic valve replacement due to regurgitation, relief of residual right ventricular outflow tract obstruction.	NA	SoV - 52 mm approximately one year before dissection	55	SoV - 70 mm, AscAo - 78 × 72mm	Stanford type A dissection from aortic root to ascending aorta	Sandwich repair of the base of the aortic root, repair of the left main stem coronary artery (compromised by the dissection flap) and an interposition tube graft (32 mm Gelweave; Terumo Aortic).	NA

M - male, F - female, NA - not available, VSD - ventricular septal defect, SoV - sinus of Valsalva, AscAo - ascending aorta, cMRI – cardiac magnetic resonance imaging, PDA – patent ductus arteriosus, *FBN1* – Fibrillin 1 gene.

There is paucity of information regarding genetic investigations in the reported patients. The patient reported by Konstantinov et al. had a diagnosis of 22q11 deletion syndrome and the Haruki et al. described a variant in the *fibrillin-1* gene (“amino acid 1128, proline was replaced with alanine”) in the individual in their report. We could not verify the pathogenicity of this variant due to the limited information provided in the manuscript.

## 4 Discussion

Here we describe a case of aortic dissection in a patient with previously operated tetralogy of Fallot and proffer a likely pathogenic variant in the *MYLK* gene as an explanation for this complication. To our knowledge, this is the first study to use whole genome sequencing and bioinformatic analysis to attempt to ascertain the genetic basis of TAD in this common congenital heart defect.

The patient had completed pregnancy approximately five months prior to TAD and had a relatively delayed TOF repair. Apart from that, the patient had no high-risk clinical characteristics for TAD identified by other studies (normotensive, female, <60 years old). Furthermore, both the aortic root and ascending aorta were only moderately dilated, and well below the threshold for preventative surgical intervention, during the last follow up before TAD [35].

The finding of a likely pathogenic variant in *MYLK* is a plausible explanation of the clinical presentation in the reported patient. The genetic variant affects an evolutionarily conserved amino-acid residue in a functional domain of the protein and therefore has potential to exert biological effect. Whilst mutations in this gene have been categorised as pathogenic in the context of TAD [22], to our knowledge, the variant described here (p.Arg1405His) has never been reported in this context. There have been four entries in ClinVar reporting the same variant, however conditions or affection status were not provided or are unknown [23]. The p. Arg1405His *MYLK* variant will require further verification before definite conclusions can be made.

*MYLK* codes for an intracellular kinase which phosphorylates myosin regulatory light chains to facilitate myosin interaction with actin filaments to produce contractile activity. Mutations in this gene have been described and validated as a causative for the development of heritable thoracic aortic disease [36,37]. In the largest reported case series, pathogenic *MYLK* variants have been reported to cause aortic dissection in aortas with minimal prior dilatation (mean SoV 39 ± 8 mm; ascending aorta 43 ± 4.9 mm) [22]. This clinical feature would fit the phenotype in the presented patient.

The finding of a genetic variant in *MYLK* in the presented patient and the search for TAD associated mutations in patients with TOF suffering TAD more generally has important clinical implications. Firstly, it may provide an explanation to the patient and cardiologist for why a rare event has occurred in a common condition and help the individual affected adjust to the life changing consequences. Secondly, the identification of pathogenic variants in this context would allow genetic cascade screening to first degree relatives. Those family members found to share the variant could then be offered serial aortic imaging and prophylactic surgical intervention as necessary. Thirdly, discoveries such as this could pave the way to establishing more detailed genetic profiles in the large population of patients with TOF who have thoracic aortic dilatation. This would identify those patients who would benefit from tailored imaging follow up and prophylactic aortic surgery.

Given the implications of this finding, it could be argued that the search for variants in genes associated with TAD should be proactively determined in patients with TOF and thoracic aortic dilatation. Further studies would be needed to ascertain the cost effectiveness of this approach. It is clearer that such studies should be conducted in patients with TOF suffering TAD as our findings demonstrate that we cannot simply ascribe TAD to TOF itself i.e., it looks likely that a proportion of these patients have independent genetic variants predisposing to TAD and therefore the TAD is an additional and independent phenotype. This would fit with our understanding of the population prevalence and genetic basis of TAD. Establishing the presence of disease-causing mutations in all patients suffering acute thoracic aortic syndromes is a cornerstone of management [35] and patients with TOF shouldn't be excluded from this practice for the reasons highlighted.

Our literature review revealed a very small number of reports of TAD in the context of TOF and TOF was present in only a small number of cases in the US National Inpatient Sample Database [9]. Of importance for the summary analysis of data is the non-uniform way the cases were reported. This is likely to originate from retrospective character of the reports as well as the time interval between the initial TOF repair and the development of TAD. The described cases show a wide spectrum of clinical phenotypes and any conclusions drawn from this cohort are affected by inadequate sample size and heterogeneity. Most of the reports provide measurement of the aorta performed after TAD. However, it has been established that the aortic dissection significantly changes the size and geometry of the aorta and the post-dissection measurements correlate poorly with those performed pre-dissection [38]. Importantly, half of the patients, for whom pre-dissection measurements have been reported, would not meet criteria for high-risk aortic dilatation and would not be offered preventative aortic surgery [35]. None of the reported manuscripts reported a patient who did not survived the TAD and therefore it is difficult to know the true incidence of TAD in those individuals with TOF to inform clinical conversations about risk.

Our findings are limited to the extent that the reported *MYLK* variant, although fitting the clinical phenotype, needs further validation by replication in another patient with TAD. We were also unable to exclude interaction of the *MYLK* variant in this patient with other genetic variants and/or environmental factors (recently completed pregnancy) in producing the clinical phenotype. Our current research ethical approval does not allow for genetic testing of the unaffected relatives of the proband and therefore we have not been able to perform sequencing of the parents of the proband to determine if the *MYLK* variant occurred de-novo. We have referred the patient to the Clinical Genetic Team, and if rs745547260 in *MYLK* is reclassified as likely-pathogenic, the relatives of our patient will undergo cascade genetic screening.

We have presented a detailed clinical description of a patient who developed TAD late after repair of TOF. To our knowledge, we have performed the first whole genome sequencing exercise in this context and identified a previously unreported and potentially pathogenic variant in the myosin light chain kinase gene. Systematically replicating this approach in patients with TOF suffering TAD will help patients or bereaved family members, as well as care givers, understand why such complications have occurred. It would allow screening of first-degree relatives, potentially saving lives by undertaking prophylactic aortic surgery in previously undiagnosed at risk individuals. Further studies assessing the logistics and cost of genetic screening in patients with TOF and thoracic aortic dilatation are needed to determine in a population based approach would have similar benefits.

## CRediT authorship contribution statement

**Radoslaw Debiec:** Writing – review & editing, Writing – original draft, Project administration, Methodology, Investigation, Formal analysis, Data curation, Conceptualization. **Armia Ebeid:** Writing – review & editing, Writing – original draft, Project administration, Methodology, Investigation, Data curation, Conceptualization. **Stephen Hamby:** Writing – review & editing, Validation, Software, Methodology, Investigation, Formal analysis, Data curation. **Odeta Anciunaite:** Writing – review & editing, Validation, Methodology, Investigation, Formal analysis, Data curation. **Anne Illsley:** Writing – review & editing, Resources, Project administration, Methodology, Investigation, Formal analysis, Data curation. **Ali Nizam:** Writing – review & editing, Project administration, Investigation, Formal analysis. **Madiha Iqbal:** Writing – review & editing, Investigation, Data curation. **Kassem Safwan:** Writing – review & editing, Supervision, Investigation, Formal analysis, Conceptualization. **Tariq Saifullah:** Writing – review & editing, Supervision, Methodology, Formal analysis, Conceptualization. **Frances Bu’Lock:** Writing – review & editing, Supervision, Project administration, Methodology, Conceptualization. **Toru Suzuki:** Writing – review & editing, Supervision, Methodology, Conceptualization. **Nilesh J. Samani:** Writing – review & editing, Supervision, Methodology, Conceptualization. **Tom Webb:** Writing – review & editing, Writing – original draft, Validation, Supervision, Project administration, Methodology, Formal analysis, Data curation, Conceptualization. **Aidan P. Bolger:** Writing – review & editing, Writing – original draft, Supervision, Resources, Project administration, Methodology, Investigation, Funding acquisition, Data curation, Conceptualization.

## Declaration of competing interest

The authors declare that they have no known competing financial interests or personal relationships that could have appeared to influence the work reported in this paper.
